# Ultrasensitive Electrochemical Biosensor for Rapid Screening of Chemicals with Estrogenic Effect

**DOI:** 10.3390/bios14090436

**Published:** 2024-09-09

**Authors:** Ruixin Li, Jin Li, Xianbo Lu, Fanli Meng, Jiping Chen

**Affiliations:** 1 CAS Key Laboratory of Separation Science for Analytical Chemistry, Dalian Institute of Chemical Physics, Chinese Academy of Sciences, No. 457 Zhongshan Road, Dalian 116023, China; 2310290@stu.neu.edu.cn (R.L.); chenjp@dicp.ac.cn (J.C.); 2 College of Information Science and Engineering, Northeastern University, Shenyang 110819, China; 3 College of Mechanical and Electronic Engineering, Northwest A&F University, Yangling 712100, China; lijin2023@nwafu.edu.cn; 4 University of Chinese Academy of Sciences, 19 Yuquan Road, Beijing 100049, China

**Keywords:** estrogen, human estrogen receptor α, estrogenic activity evaluation, electrochemical biosensor, endocrine disrupting chemicals (EDCs)

## Abstract

Estrogenic chemicals are widely distributed and structurally diverse. They primarily disrupt estrogen-related metabolism in animals or humans by mimicking the agonistic receptor effects of natural estrogens, thereby influencing the transcription of estrogen receptors to regulate their quantity and sensitivity. This disruption of estrogen-related metabolism can lead to estrogen-related effects, posing risks to biological health, emphasizing the urgent need for simple and effective methods to screen compounds with estrogenic effects. Herein, a new electrochemical biological effect biosensor based on human estrogen receptor α (hERα) is developed, which uses hERα as the biorecognition element and employs the electroactive horseradish peroxidase (HRP) labeled 17β-estradiol (E2) multifunctional conjugate HRP-E2 as the signal-boosting element and ligand competition agent. Based on the specific ligand–receptor interaction principle between the target and nuclear receptor, by allowing the test compound to compete with HRP-E2 conjugate for binding to hERα and testing the electrocatalytic signal of the conjugate that fails to bind to the hERα estrogen receptor, rapid screening and quantitative detection of chemical substances with estrogenic effect have been achieved. The biosensor shows a wide linear range of 40 pM to 40 nM with a detection limit of 17 pM (S/N = 3) for E2, and the detection limit is 2 orders of magnitude better than that of the previously reported sensors. The biosensor based on ligand–receptor binding can not only quantitatively analyze the typical estrogen E2, but also evaluate the relative estrogen effect strength of other estrogen compounds, which has good stability and selectivity. This electrochemical sensing platform displays its promising potential for rapid screening and quantitative detection of chemicals with estrogenic effects.

## 1. Introduction

Environmental hormones are considered to be the third environmental problem after global warming and ozone layer destruction. In recent years, an increasing number of environmental chemical substances have been shown to exhibit estrogenic effects. Estrogenic chemicals have been found in wastewater, surface water, and even drinking water worldwide [1]. They can enter the organism via pathways such as ingestion via the digestive tract, inhalation via the respiratory system, and absorption via the skin, and affect biological health by interfering with the endocrine system, binding to hormone receptors, and/or regulating gene expression [2,3]. These environmental hormones have structures similar to natural hormones and can mimic the actions of natural estrogens, affecting the activity and function of estrogen receptors. These interferences can disrupt estrogen-related metabolism in animals or humans, further endangering biological health [4]. These environmental hormones may lead to health issues such as obesity [5], thyroid secretion abnormalities [6], reproductive system problems like gonadal developmental abnormalities [7], estrogen-dependent tumors [8] such as breast cancer [9], endometrial cancer [10], neuroblastoma [11], melanoma [12], and neurological behavioral disorders [13] such as autism spectrum disorders [14] and attention deficit hyperactivity disorder [15]. Although these chemicals are present in low concentrations in the environment, their harm to organisms is still significant.

With the increasing global concern about the adverse effects of environmental estrogens on human health, there is an urgent need to develop a method for screening and detecting chemicals with estrogenic effects. Currently, various methods have been reported for the detection of environmental estrogen pollutants. Traditional detection methods mainly include chromatography/mass spectrometry analysis [16,17], enzyme-linked immunosorbent assay [18,19], and capillary electrophoresis [20]. Although these methods are highly specific and sensitive, they often suffer from issues such as lack of portability of equipment, high costs, tedious and time-consuming sample pretreatment, and the need for specialized personnel to operate.

A biosensor is a type of sensory device used to collect information on certain biophysical or chemical changes and then convert this information into measurable signals. An ideal biosensor should not only have the ability to accurately identify target substances but also be able to respond to analytes at low concentrations. They are often modified with enzymes, antibodies, and oligonucleotide biomolecules to enhance their selectivity [21]. In the field of estrogen compound detection, the emergence of biosensors complements traditional detection methods, allowing for high sensitivity and strong specificity in identification, while also enabling simpler instrument operation, faster signal response, and cheaper testing costs. These advantages have led to their wide application in various fields such as medicine and the environment. In 2019, Liu et al. developed an estrogen receptor (ER)-based evanescent wave fluorescent biosensor by using a triple functional small-molecule–protein conjugate as a signal probe for the determination of estrogenic activities in water samples, and the biosensor achieved a detection limit of 1.05 μg/L (E2 equivalent concentration) [22]. In 2023, Tan et al. developed a conformation-specific reporter-mediated fiber optic evanescent wave biosensor, which was used to screen potential estrogenic agonists and antagonists in wastewater samples [23]. These cutting-edge optical sensors have been used to screen chemicals with estrogenic activity, but there are still some problems that need to be solved, such as susceptibility to various interferences, large equipment size, and the need to improve detection limits.

Electrochemical biosensors are analytical detection devices that combine biological recognition processes with electrochemical sensing technology. They harness the delicate sensitivity and specificity of biological systems to provide measurements in a simple and user-friendly format. With small size, light weight, and portability, electrochemical devices enable efficient and cost-effective online and on-site detection of pollutants, characteristics that traditional large-scale instruments lack [24]. Currently, electrochemical biosensors used for estrogen detection mainly employ enzymes [25,26,27], aptamers [28,29,30], molecularly imprinted polymers [31,32,33], and antibody/antigens [34,35,36] as biorecognition elements. Although detection methods based on these biological recognition mechanisms can detect a known or established estrogen compound, they are unable to screen whether known or unknown compounds possess estrogenic effects. It is also impossible to compare the potential estrogenic effect intensities (EEI) of estrogen compounds by using these conventional biosensors.

Human estrogen receptor α (hERα) as a member of the nuclear receptor superfamily, is the primary target of estrogen capable of recognizing all chemical substances with estrogenic activity. Herein, we developed an electrochemical biosensor utilizing hERα as the biorecognition element for rapid screening and detection of estrogens, and assessment of their relative estrogenic effects. A multifunctional conjugate of E2-HRP was designed to serve as the signal-boosting element and ligand competition agent. Based on the specific ligand–receptor interaction principle between the target and estrogen receptor, the E2 moiety of the E2-HRP conjugate competes with the estrogenic targets that can bind to hERα and leave the unbound E2-HRP conjugates to be separated and immobilized on the working electrode surface. Subsequently, electrochemical differential pulse voltammetry (DPV) was performed in the buffer solution containing tetramethylbenzidine (TMB) and hydrogen peroxide and obtained two distinctive oxidation peaks of TMB. Because the HRP moiety of the E2-HRP conjugate on the modified electrode can catalyze TMB in the buffer solution, it results in a decrease in the DPV peak currents of TMB. Therefore, the higher the concentration and estrogenic effect intensities of the estrogenic chemical measured, the higher the remaining unbound E2-HRP conjugate concentration, leading to more TMB being catalyzed to oxidized TMB by E2-HRP. Consequently, the electrochemical sensing signal of TMB weakens, showing an inverse relationship between the intensity of the electrochemical signal and the concentration/EEI of the target being measured. This biosensing method demonstrates high sensitivity, good reproducibility, long-term stability, and a high recovery rate for the detection of estrogen, offering wide application prospects in estrogenic substance detection and estrogenic effect screening.

## 2. Experimental

### 2.1. Materials and Apparatus

Beta-estradiol 17-semisuccinate (E2-COOH) and 1-(3-dimethylaminopropyl)-3-ethylcarbodiimide hydrochloride (EDC) were purchased from Merck Chemical Technology Co., Ltd. (Shanghai, China). Bisphenol A (BPA), N-hydroxysuccinimide (NHS), and methanol were purchased from Maclin Biochemical Material Technology Co., Ltd. (Shanghai, China). Potassium chloride (KCl), Sodium chloride (NaCl), potassium dihydrogen phosphate (KH_2_PO_4_), disodium hydrogen phosphate dodecahydrate (Na_2_HPO_4_∙12H_2_O), and hydrogen peroxide (H_2_O_2_, 30% *v*/*v*) were purchased from Damao Chemical Reagent Factory (Tianjin, China). Horseradish peroxidase (HRP) was obtained from Boer Chemical Reagent Co., Ltd. (Shanghai, China). Anhydrous citric acid and anhydrous ethanol were purchased from Bonuo Biotechnology Reagent Factory (Dalian, China). Human estrogen receptor protein (hERα) and 17β-estradiol (E2) were purchased from Nuofan Biological Co., Ltd. (Beijing, China) and Tongming Weiye Technology Development Co., Ltd. (Beijing, China), respectively. TMB, Estrone (E1), estriol (E3), and testosterone (Te) were purchased from Aladdin Bio-Chem Technology Co., Ltd. (Shanghai, China). Triiodothyronine (T3) was from Zeye Biological Technology Co., Ltd. (Shanghai, China). The Ni-NTA-agarose resin was from BBI Life Sciences Corporation (Beijing, China). Milli-Q water with 18 MΩ cm (Millipore, Burlington, MA, USA) was used throughout all experiments. UV-Vis absorbance spectra were performed using a UV spectrophotometer (Mettler Toledo, Zurich, Switzerland). Scanning electron micrograph (SEM) was taken with a JSM7401 scanning electron microscope (JEOL Ltd., Tokyo, Japan). All electrochemical tests were conducted using a portable PalmSens4 working station (Palmsens B.V., Houten, Netherlands). Electrochemical DPV testing was conducted in a TMB substrate solution with a scan range from 0.0 V to 0.8 V at a scan rate of 0.05 V/s.

### 2.2. Preparation of Solution Needed for the Experiment

The TMB substrate solution is a 50 mM citric acid–sodium phosphate buffer at pH 5.0 containing 0.2 mM TMB and 0.04% (*v*:*v*) H_2_O_2_. The E2-COOH stock solution was prepared in methanol at a concentration of 2 mM and then diluted to 1 mM with phosphate-buffer solution (PBS) at pH 5.5 for later use. EDC and NHS were dissolved separately in 10 mM PBS buffer at pH 5.5, both at a concentration of 12 mM. The HRP solution was prepared in 10 mM PBS buffer at pH 7.4, with a concentration of 10 mg mL^−1^.

### 2.3. Preparation of E2-HRP Conjugate

The conjugation of E2-HRP is based on the principle of the formation of an amide bond via the reaction between carboxyl (-COOH) and amino (-NH_2_) groups. HRP is essentially a protein formed by the dehydration condensation and folding of multiple amino acids, providing a certain number of free amino groups on its surface for conjugation reactions. E2-COOH is a derivative of E2, with a structure that replaces the hydroxyl group on the original E2 structure with a carboxyl group connected by a four-carbon chain. The structural diagrams of E2 and E2-COOH are shown in Supplementary Materials Figure S1.

Take 400 μL of E2-COOH solution and add 100 μL each of EDC and NHS solutions; this step is to activate the carboxyl group structure of E2-COOH into an amide intermediate that can react with amino groups. After 2 h, slowly add 400 μL of HRP solution directly to the mixed solution, and then incubate for 4 h to allow full reaction between HRP and E2-COOH to generate the E2-HRP conjugate. To ensure a thorough reaction of HRP, an excess of E2-COOH can be added during the conjugate preparation. After the conjugate is formed, purification can be performed to remove any unreacted E2-COOH. The purification process involves dialysis, in which the E2-HRP conjugate has a molecular weight exceeding 40,000 Da, and the dialysis membrane used has a pore size allowing only small molecules with a molecular weight below 20,000 Da to pass through, thereby removing unreacted E2-COOH (molecular weight 372.45), EDC (molecular weight 98.96), and NHS (molecular weight 115.09) from the E2-HRP solution. After dialyzing in a 1 L volume of PBS (pH 7.4) for 4 h, the remaining liquid inside the dialysis tubing is withdrawn using a pipette and adjusted to a volume of 4 mL (at this point, the final concentration of E2-HRP, based on HRP standards, is 1 mg/mL). The conjugate should be sealed, protected from light, and stored in a refrigerator at 4 °C for future use.

### 2.4. Experimental Protocol Design

**Figure 1:**
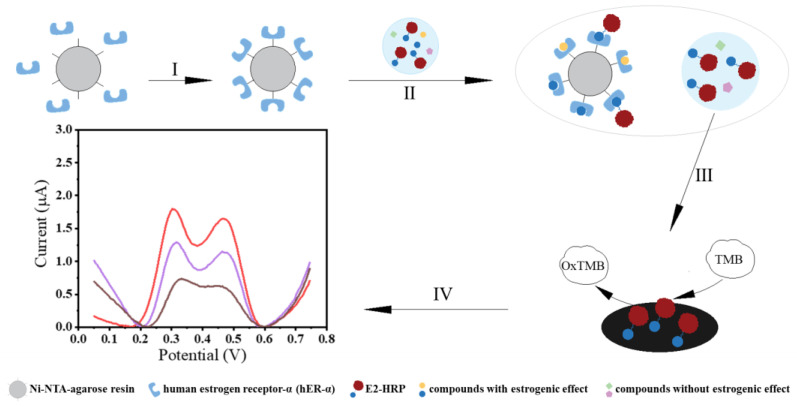
Schematic diagram of the sample preparation (**top row**) and biosensing detection (**bottom row**). (I) Incubate for 2 h to allow full binding of hER-α with Ni-NTA-agarose resin; (II) Add E2-HRP and the compound to be tested then incubate for 1 h; (III) Centrifuge at 3000 rpm for 3 min then take the supernatant and modify it on the GCE electrode; (IV) Electrochemical biosensing signals boosted by electrocatalytic reaction between E2-HRP and TMB substrate solution.

The purification of hERα in the experiment is carried out using an HIS tag, which can bind to the Ni^2+^ ions on the surface of Ni-NTA-agarose resin, thereby immobilizing hERα on the Ni-NTA-agarose resin surface. Figure 1 depicts the experimental protocol for the electrochemical biosensing method in this work, which was based on the ligand-estrogenic receptor interaction for estrogenic activity evaluation of chemicals or actual samples. In brief, an E2-HRP conjugate composed of HRP and E2 moiety was designed, which competes with estrogenically active chemical substances in the test sample to bind to hERα immobilized on the Ni-NTA-agarose resin surface. When there are more estrogenically active chemical substances in the test sample, more E2-HRP conjugates will not be able to bind to hERα and will remain free in the solution. After centrifugation separation, the unbound E2-HRP conjugates are isolated and 3 μL supernatant is immobilized on the working electrode surface by 2 μL 0.5% Nafion solution. Herein, the E2-HRP conjugate serves as a signal transducer element. The HRP in the conjugate can catalyze the oxidation of TMB to OxTMB (oxidized TMB) in the TMB substrate solution in the presence of H_2_O_2_. Typically, the stronger the estrogenic activity of the target or actual sample, the more residual E2-HRP conjugates remain, leading to a higher amount of oxidized TMB. Consequently, the electrochemical DPV signals for TMB decrease as the estrogenic activity increases. As shown in Figure 1 (IV), there are two distinctive responsive peaks in the cyclic voltammograms, which correspond to the electrochemical signals boosted by the electrocatalytic reaction between the E2-HRP and TMB substrate solution. It means that each target is detected by two electrochemical response signals. In this study, the sum of the two DPV peak current signals was used as a y-axis to establish a calibration curve for targets.

## 3. Results and Discussion

### 3.1. Verification of E2-HRP Conjugate Activity

**Figure 2:**
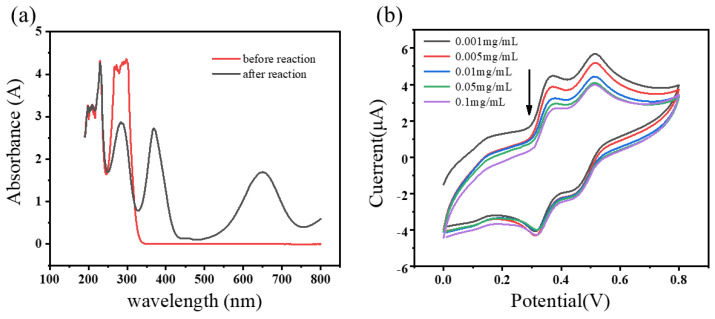
(**a**) UV-Vis absorption spectra of TMB substrate solution (containing 0.2 mM TMB and 0.04% (*v*:*v*) H_2_O_2_) before and after the catalytic reaction of E2-HRP (1 mg/mL) for TMB. (**b**) The electrochemical cyclic voltammograms of the TMB substrate solution after the addition of various concentrations of E2-HRP.

The catalytic activity of E2-HRP was first verified using UV-Vis spectroscopy. An amount of 5 mL of TMB substrate solution was mixed with 1 μL of 4 μg/mL E2-HRP conjugate solution, and after a 5 min reaction, the UV absorption spectrum was measured using a UV-Vis spectrophotometer. The results before and after the reaction are shown in Figure 2a. There are distinct differences in the UV absorption peaks at 380 nm and 650 nm before and after the reaction, attributed to the rapid catalysis of E2-HRP for TMB, leading to the oxidation of TMB to OxTMB in the solution, causing the change in the absorption peaks.

The catalytic activity of E2-HRP was further verified using electrochemical methods. Figure 2b shows the CV curves (ranging from 0.0 V to 0.8 V) obtained after sequentially adding 2 μL of 0.001, 0.005, 0.01, 0.05, and 0.1 mg/mL of E2-HRP into the TMB substrate solution. It can be observed that the CV oxidation peak decreases as the concentration of E2-HRP increases. This phenomenon occurs because when E2-HRP conjugates are introduced into the reaction system, TMB is oxidized by the HRP in the conjugate. The more TMB is catalyzed for oxidation by HRP, the less TMB is available to participate in the electrochemical oxidation process, resulting in a decrease in the oxidation signal.

Both UV-Vis spectroscopy and electrochemical methods have demonstrated the excellent catalytic activity of the E2-HRP conjugate synthesized in our study, making it suitable for subsequent experiments.

### 3.2. Feasibility Analysis of the Experimental Approach

The feasibility of the experimental plan was verified by using UV-Vis spectrophotometry and electrochemical methods, respectively.

**Figure 3:**
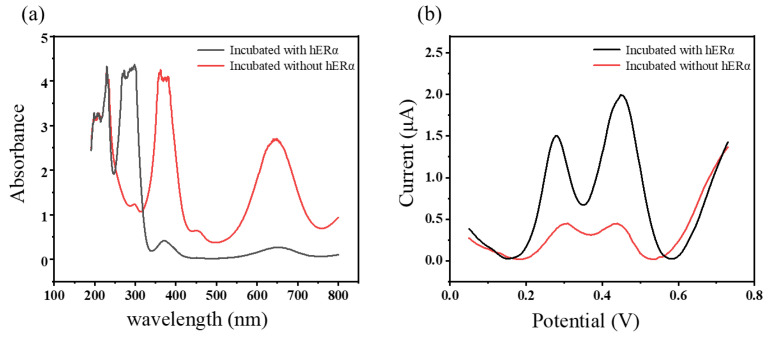
(**a**) The UV absorption spectra of the TMB substrate solution (containing 0.2 mM TMB and 0.04% (*v*:*v*) H_2_O_2_) after reaction with supernatants incubated in the presence (red) or absence (black) of 1mg/mL hERα. (**b**) The DPV curves of the different supernatants (incubated with or without hERα) modified electrodes for the TMB substrate solution.

Firstly, experiments were conducted according to the procedures in Figure 1 under conditions with or without the co-incubation of hERα. After the experiments concluded, 1 μL of supernatant was taken from each and added to 1 mL TMB substrate solution, the reaction was allowed to proceed for 3 min before testing the UV-Vis absorption spectra of both, with the results shown in Figure 3a. From the spectral data, it can be observed that there are significant differences in the UV absorption peaks at 380 and 650 nm for experiments conducted with or without the co-incubation of hERα. This difference is attributed to the ligand–receptor interaction between E2-HRP and hERα, wherein the presence of hERα co-incubation, most of the E2-HRP is captured by hERα, resulting in a minimal content in the supernatant, consequently a reduced catalytic effect on the TMB substrate solution. In experiments conducted without the co-incubation of hERα, E2-HRP does not have a specific target to bind to and is mostly present in the supernatant; this allows for the catalysis of more TMB substrate solution, leading to significantly enhanced characteristic absorption at 380 and 650 nm in the UV absorption spectra. In summary, under conditions with or without the co-incubation of hERα, adding the supernatant to the TMB substrate solution and observing the differences in UV absorption peaks after the reaction, demonstrates that the prepared E2-HRP conjugate can specifically bind to hERα.

Electrochemical experiments were conducted to further verify the feasibility of the experimental approach by adding E2-HRP under conditions with or without the co-incubation of hERα. After incubation, 3 μL of supernatant was taken from each and modified on the electrode surface, the electrodes were then dried, and the supernatant was fixed on the electrode surface using 2 μL Nafion solution (0.5% *v*/*v*). DPV signals were measured separately, and the results are shown in Figure 3b. Figure 3b shows significant differences in signal intensities between experiments conducted with or without the co-incubation of hERα, this is because when hERα is co-incubated, it captures E2-HRP in the solution system, resulting in a lower amount of E2-HRP in the supernatant, consequently, the quantity of E2-HRP modified on the electrode surface is minimal, leading to a weaker catalytic effect and DPV signal on the TMB substrate solution. In experiments conducted without the co-incubation of hERα, the results are exactly opposite to those with hERα co-incubation, demonstrating that the prepared E2-HRP conjugate can specifically bind to hERα.

### 3.3. Optimization of Experimental Conditions

Due to the fact that 1 mL of Ni-NTA-agarose resin can bind at least 10 mg of His-tagged protein, while E2-HRP can bind to hERα at a maximum molar ratio of 1:1, in order to achieve the best performance, it is necessary to optimize the amounts of various reagents and materials used in the experiment. However, for practicality considerations, during actual experimental procedures, a minimum of 2 μL of solution needs to be taken each time to ensure stable and repeatable experiments. Therefore, the quantities of Ni-NTA-agarose resin, hERα, and E2-HRP are determined to be 2 μL, 3 μL, and 2 μL, respectively. After incubation, it is necessary to separate the Ni-NTA-agarose resin-hERα-ligand complex from the unbound free E2-HRP by centrifugation and extract the supernatant using a pipette. The addition volume of the test compound is crucial, aiming to minimize the volume of the solution while ensuring that the supernatant extracted is free from impurities (Ni-NTA-agarose resin–hERα-ligand complex). Via multiple centrifugation attempts, a final decision is made to add 50 μL of the test compound for the incubation experiment.

The final concentration of Nafion is determined via optimization experiments, where five volume concentrations of Nafion solution (0.05, 0.1, 0.5, 1.0, and 1.5%) are selected for 5 sets of experiments. Each concentration of Nafion solution is used according to the experimental protocol, with steps involving testing with 0 nM and 40 nM of E2 as competitive binding substances to E2-HRP. This setup allows obtaining electrochemical signals for each Nafion concentration corresponding to two different concentrations of E2. By dividing the electrochemical signal value obtained at 0 nM by that obtained at 40 nM, a signal amplification factor is obtained. To achieve higher sensitivity and a wider detection range in subsequent estrogen screening and detection experiments, the concentration of Nafion corresponding to the data set with the highest signal amplification factor needs to be selected. The signal amplification factor chart from the Nafion concentration optimization experiment is shown in Supplementary Materials Figure S2, and the optimized concentration of the Nation solution is 0.5%. At this optimized Nafion concentration, the E2-HRP and Nafion composite can form a stable and compact film on the electrode, as shown in Supplementary Materials Figure S3.

### 3.4. Response Characteristics of the Biosensor for Typical Estrogen E2

**Figure 4:**
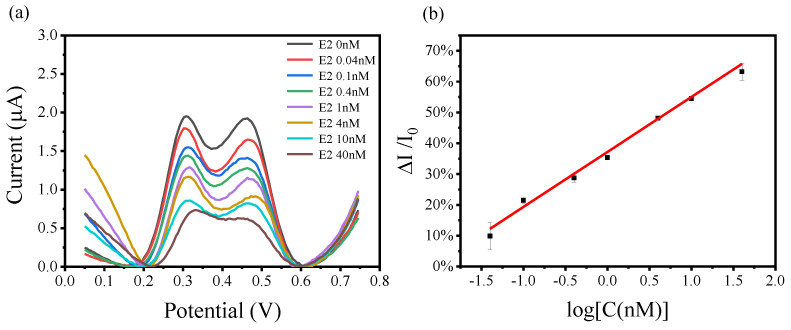
(**a**) The electrochemical DPV curves of the biosensor for different concentrations of E2. (**b**) The linear fitting curves of the response current change (%) vs. the logarithmic concentrations of E2.

E2 is the most important endogenous estrogen, which is used to study the response characteristics and to verify the performance of the developed biosensor. The typical DPV response curves of the biosensor for different concentrations of E2 ranging from 0 nM to 40 nM are shown in Figure 4a. It can be seen that the response currents of the biosensor decrease with increasing concentrations of E2. Figure 4b shows the corresponding calibration curve of the biosensor for E2 detection. During the calculation, the sum of two peak current values is taken for each curve, and each data point on the calibration curve represents the average signal of three tests. It is obvious from Figure 4b that there is a linear relationship between the signal intensity of electrocatalysis for TMB and the concentrations of E2. When the E2 concentration is 0 nM, the TMB oxidation signal is the strongest, and the TMB oxidation signal gradually decreases as the E2 concentration increases. In the concentration range of E2 from 0.04 nM to 40 nM, the response currents of the biosensor show a linear relationship for the logarithmic concentration of E2 (y = 0.3718x + 0.1784, R^2^ = 0.9853) with an excellent limit of detection (LOD) of 17 pM. The sensor demonstrates high sensitivity and good linearity in response to E2. The LOD of this sensor for E2 is 2 orders of magnitude better than that of the electrochemical sensors recently reported in the literature. For example, Xie et al. developed a sensitive electrochemical sensor based on wrinkled mesoporous carbon nanomaterials for rapid and reliable assay of E2, and the sensor achieved a minimum detection limit of 8.3 nM for E2 [37]. Da Silva et al. developed an electrochemical sensor modified with a molecularly imprinted polymer and carbon black for E2 detection, and this sensor’s minimum detection limit for E2 can reach as low as 30 nM [38].

### 3.5. Response Characteristics of the Biosensor for Other Compounds

To investigate the selective recognition capability of the biosensor for estrogenic compounds, in addition to E2, three representative estrogenic compounds (estrone, estriol, bisphenol A) and two typical androgenic compounds (testosterone, triiodothyronine) were individually tested by the electrochemical biosensor. The concentration of the chosen interferents was set to be the same as that of the primary target, E2. Two representative levels—one high (10 nM) and one low (1 nM)—were selected within the sensor’s linear range, as the concentration of these interferents is usually at this level. By comparing the electrochemical signals with those of the typical estrogen E2 at the same concentrations (or at the same response currents), the sensor’s selective recognition capability for estrogenic compounds was analyzed. Theoretically, the stronger the estrogenic effect of the measured compound and the higher the concentration, the greater the change in the electrochemical DPV peak current. The relative estrogenic effects intensities of different target substances can be evaluated by comparing the magnitude of the response signal changes on the sensor for different target substances at the same concentration, or by comparing the concentrations of different target substances that cause the same signal change on the sensor.

**Figure 5:**
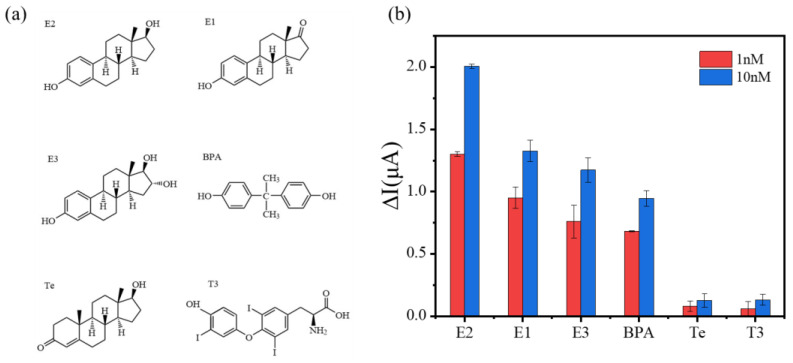
(**a**) Structures of E2, E1, E3, BPA, Te and T3. (**b**) The response signal comparison chart of E2, E1, E3, BPA, Te, and T3 at concentrations of 1nM and 10nM.

The structural diagrams of E2, E1, E3, BPA, Te, and T3 are shown in Figure 5a, while the comparative electrochemical response signals of these compounds are illustrated in Figure 5b. As depicted in Figure 5a, the tested estrogenic and androgenic compounds exhibit similar chemical structures, all containing basic structural elements of a benzene ring and a phenolic hydroxyl group. However, the electrochemical detection results of these compounds show significant differences. As shown in Figure 5b, at the same concentration, E2, E1, E3, and BPA exhibit noticeable signal responses, but the response intensities show a decreasing trend. Although E2, E1, and E3 are endogenous estrogens naturally secreted by the human body, E2 has the highest intensity and the strongest activity, while E1 and E3 are metabolites of E2, leading to a pronounced change in electrochemical signal compared to E2. BPA, compared to E2, E1, and E3, is a typical exogenous estrogen. Exogenous estrogens, although they can bind to estrogen receptors affecting biological functions, have slightly weaker estrogenic activity compared to endogenous estrogens, resulting in a lower electrochemical signal response of the biosensor for BPA than for E2, E1, and E3. Ultimately, the estrogenic effect of these four estrogenic compounds is ranked as E2 > E1 > E3 > BPA, a conclusion consistent with previous research [39]. At the same concentration, Te and T3 both exhibit minimal negligible response signals, although their structures are extremely similar to typical estrogens, revealing the good selectivity and specificity of the electrochemical biosensor for estrogenic compounds.

### 3.6. Reproducibility and Stability of the Biosensor

Ten individual preparations and tests of the biosensors were conducted with a 10 nM E2 concentration, and the calculated results yielded an RSD of 2.3%, indicating good reproducibility of the biosensor. Using a similar experimental procedure, the modified working electrode was stored in a 4 °C refrigerator for one week before electrochemical testing, and the electrochemical response signal retained 96% of its original value, demonstrating good stability of the biosensor. The reproducibility and stability data of the biosensor are shown in Supplementary Materials Figures S4 and S5.

### 3.7. Actual Sample Testing

To further evaluate the reliability of the electrochemical biosensor, estrogenic effect screening tests were conducted by standard recovery test on two actual samples, tap water and mineral water. The tap water sample was obtained from the laboratory, while the mineral water sample was purchased from a local supermarket. Equal volumes of PBS buffer solution were added to the tap water and mineral water samples, mixed well, and then blank and fortified experiments were conducted accordingly. The experimental results are shown in Table 1. The biosensor displays excellent recoveries ranging from 92% to 106% in the detection of actual water samples.

## 4. Conclusions

This study successfully developed an electrochemical nuclear receptor biosensor for estrogen screening and quantification. It operates via competitive binding between the E2-HRP conjugate and the analyte with human estrogen receptor α. The electrochemical hERα-based biosensor employs electroactive HRP-labeled E2-HRP conjugate as signal-enhancing elements. The electrochemical signal generated by the uncombined E2-HRP catalyzing the TMB substrate solution serves as a reference standard for sensitive quantitative or qualitative analysis of the analyte. This biosensor can quantify not only typical estrogens but also assess the estrogenic effect intensity of emerging chemicals using E2 as the reference. By converting the overall estrogenic activity level of multiple co-existing analytes in actual samples into the equivalent concentration of E2 (the naturally occurring important estrogen in the human body), the biosensor enables the quantification of estrogen activity of multiple analytes simultaneously. Importantly, this developed biosensing method serves as a crucial biological effects sensor, complementing traditional analysis and sensing methods. The biosensor exhibits excellent sensitivity, stability, selectivity, and reproducibility, with broad application prospects for quantitatively detecting known estrogens and screening the potential estrogenic effects of known or unknown chemicals and actual samples.

## Figures and Tables

**Table 1:** Standard recovery experimental results of tap water and mineral water samples by the developed biosensor.

Sample	E2 Spiked Concentration	Actual Detected Concentration	Recovery
Tap water	0 nM	0 nM	100%
	1 nM	1.06 nM	106%
	10 nM	9.20 nM	92%
Mineral water	0 nM	0 nM	100%
	1 nM	0.95 nM	95%
	10 nM	10.2 nM	102%

## Data Availability

Data will be made available on request.

## Supplementary Materials

The following supporting information can be downloaded at https://www.mdpi.com/article/10.3390/bios14090436/s1. Figure S1: Structures of E2 (17β-estradiol) and E2-COOH; Figure S2: Signal magnification factor vs. different concentrations of Nafion on the biosensor. Figure S3: Scanning electron micrograph of the E2-HRP and Nafion (0.5%) composite film modified on the electrode. Figure S4: The reproducibility data chart of the biosensor. Figure S5: The stability data chart of the biosensor.
