# Supplementary material for: Ultrasensitive Electrochemical Biosensor for Rapid Screening of Chemicals with Estrogenic Effect
Source: Biosensors (Basel). 2024 Sep 9;14(9):436. doi: 10.3390/bios14090436 (PMC11430529; doi:10.3390/bios14090436)
Supplement: Supplementary file 1 [file biosensors-14-00436-s001.zip › biosensors-3136934-supplementary.pdf]

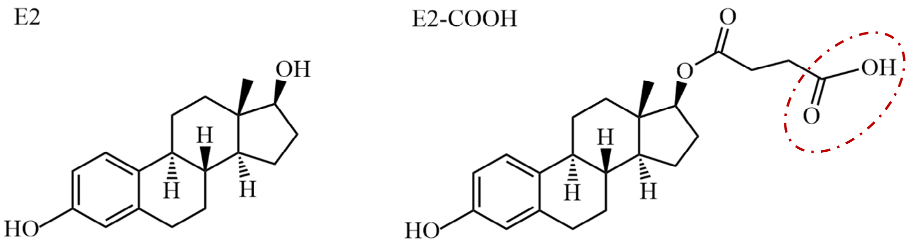

Figure S1. Structures of E2 (17β-estradiol) and E2-COOH.

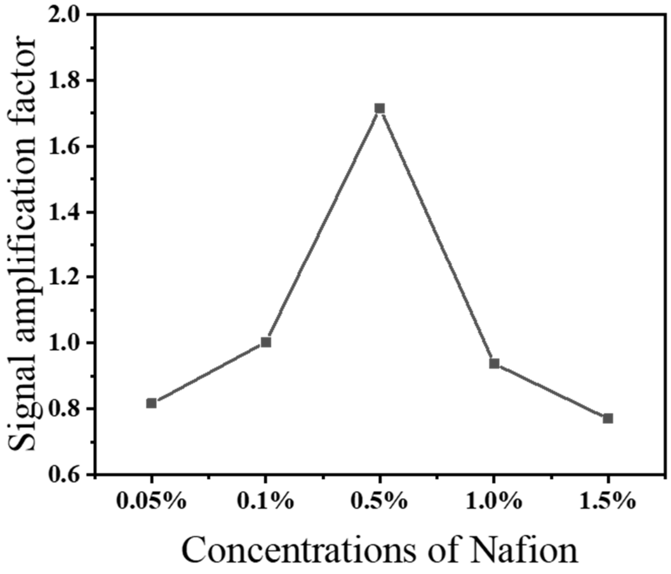

Figure S2. Signal magnification factor vs. different concentrations of Nafion on the biosensor.

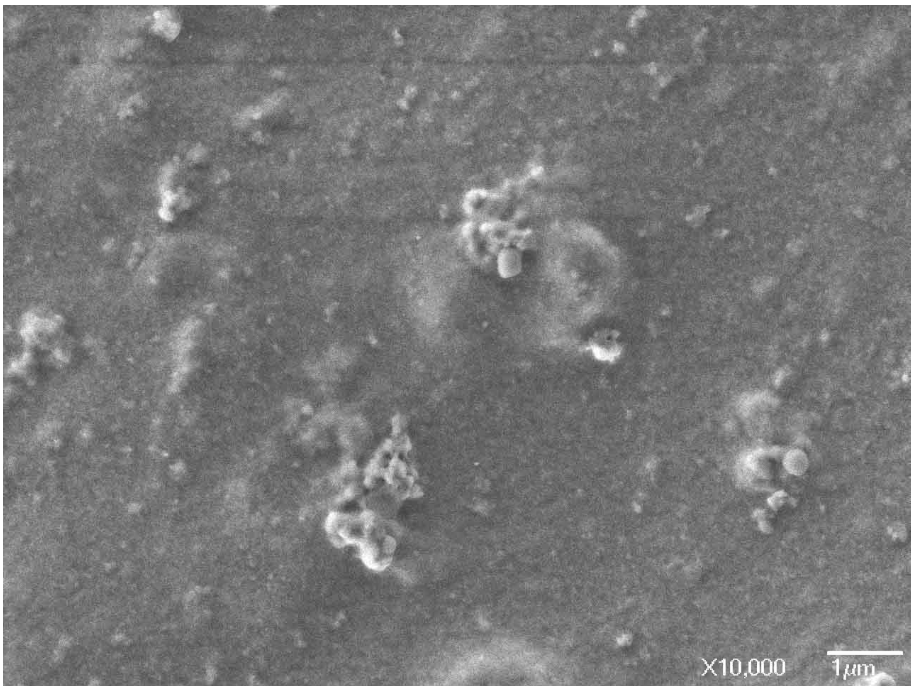

Figure S3. Scanning electron micrograph of the E2-HRP and Nafion (0.5%) composite film modified on the electrode.

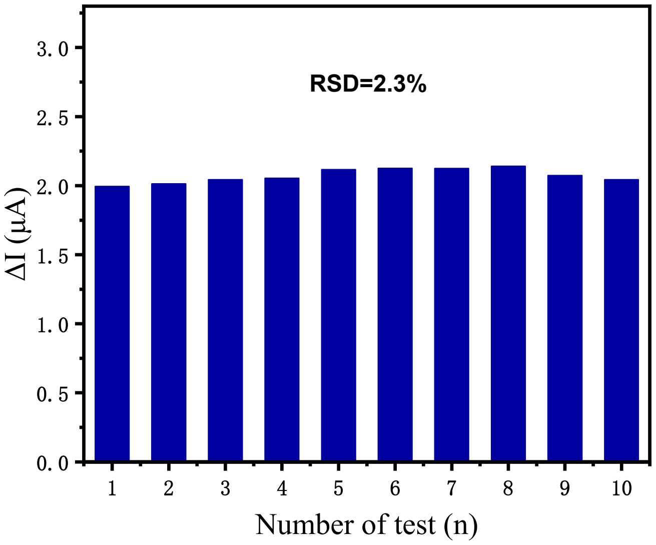

Figure S4. The reproducibility data chart of this work.

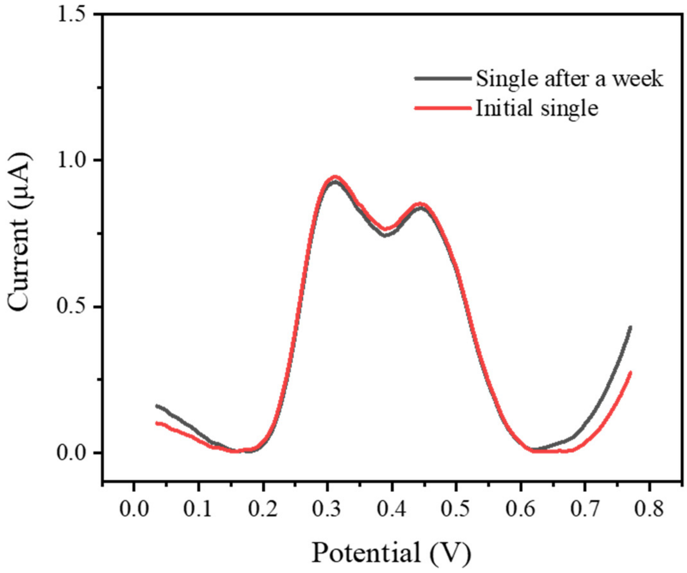

Figure S5. The stability data chart of this work (The red line represents the signal obtained immediately after preparing the electrode, while the black line represents the signal measured after storing the prepared electrode at 4°C for one week).
